# A UNIQUE MODEL TO ADDRESS ELDER ABUSE: THE OHIO ATTORNEY GENERAL’S ELDER ABUSE COMMISSION

**DOI:** 10.1093/geroni/igz038.2130

**Published:** 2019-11-08

**Authors:** Georgia Anetzberger, Amy Restorick Roberts

**Affiliations:** 1 Case Western Reserve University, Cleveland, Ohio, United States; 2 Miami University, Oxford, Ohio, United States

## Abstract

This presentation will highlight Ohio’s innovative model to address the prevalence, risk factors, and consequences of elder abuse and neglect. We will begin with an overview of the mission and duties of the recently codified Ohio Attorney General’s Elder Abuse Commission. These include: (1) to raise awareness, improve education, and boost the level of research regarding elder abuse in Ohio, (2) to provide a forum for improving the elder justice system, and (3) to identify policy, funding, and programming recommendations to address elder abuse. Next, we will share findings from the Research Committee’s first statewide survey on elder abuse research priorities. Completed by frontline practitioners, program administrators, advocates, researchers, and policy makers, the online survey received 459 responses from individuals across the state. Findings reflect gaps in the elder abuse literature, as respondents prioritized funding for research in how elder abuse can be prevented, and what programs/policies can best serve victims and their families after elder abuse has begun. We will conclude with recommendations regarding how the Elder Abuse Commission model may be adapted or modified to elevate awareness of elder abuse and elder abuse research in other parts of the country.

